# International norm development and change: can international law play a meaningful role in curbing the lifestyle disease pandemic?

**DOI:** 10.1186/s12914-020-00239-7

**Published:** 2020-07-23

**Authors:** Preslava Stoeva

**Affiliations:** Department of Global Health and Development, London School of Hygiene and Tropical Medicine, 15-17 Tavistock Place, London, WC1H 9SH UK

**Keywords:** Norm development, Agentic constructivism, Critical legal studies, Noncommunicable diseases, Politics, International legal principles, International relations

## Abstract

**Background:**

The magnitude of the noncommunicable epidemic is difficult to overstate. The projected cost of the epidemic is substantial. It disproportionately affects people in low- and middle-income countries as well as poorer and marginalised communities in high-income countries. The international community has taken various steps to address the four modifiable risk factors causing the majority of noncommunicable diseases (NCDs), however, action has so far fallen short of expectations. Both analysts and international institutions are advocating the adoption of a new international legal norm to address the NCD crisis.

**Main text:**

Drawing on existing knowledge from international relations and international legal studies, this article argues that a new international treaty is not only currently improbable, but also not strictly desirable. In-depth critical analysis and reflection is needed regarding the strengths and weaknesses of a legal approach to addressing the NCD pandemic. The argument is set out in three sections - the first reviews contributions of agentic constructivism, which focus on the *process* of normative emergence and change, and draws on empirical examples to highlight overlooked aspects of normative development and how they relate to NCD politics. The second engages with the critique of legal *principles*. Critical approaches to law seek to expose the myths that legal principles are neutral, objective, good. The third section discusses the characteristics of *practice* in the NCD field and its implications on process and principles for the pursuit of a legal solution to the NCD crisis.

**Conclusions:**

Any advocacy for an international norm to address NCDs needs to be nuanced and demonstrate awareness of the nature and character of both the norm development process and resulting international legal principles. As analysts, we are responsible for advocating inclusive and ethical norms, but also for highlighting the implications of inequalities and differences between and within states and societies. There may be a viable international legal instrument that would support dedicated policies to curb the NCD epidemic, but such an instrument needs to be actively advocated for and negotiated with a wide range of stakeholders, navigating a complex international framework of existing norms and conflicting, powerful interests.

## Background

‘Noncommunicable diseases (NCDs) are the leading cause of death globally and one of the major challenges of the 21st century’ (WHO [91]: 10). An estimated 71% of all deaths globally in 2016 resulted from NCDs, the World Health Organisation (WHO) reports. Over the next 20 years NCDs will cost more than USD 30 trillion, pushing millions of people below the poverty line ([6]: 5). The four major NCDs are cardiovascular diseases, cancers, chronic respiratory diseases and diabetes (WHO [91]: 10) and the four modifiable risk factors associated with these - tobacco, alcohol, poor diet and physical inactivity. It is worth emphasising here that there has been no shortage of international action on NCDs over the last two decades - the international community adopted the Framework Convention on Tobacco Control (2003) and its Optional protocol (2012), committed to taking action to prevent and control NCDs at three United Nations General Assembly High-Level Meetings (2011, 2014, 2018), and the WHO produced a number of action plans and strategies for addressing the modifiable causes of NCDs - tobacco use, harmful use of alcohol, diet and physical inactivity ([92]: 9–10). As will be discussed below, there is a significant and expanding body of literature on different aspects of the noncommunicable disease crisis, which, most agree, has reached pandemic proportions [1]. The crisis is real and projections estimate it is likely to get worse, due to a combination of factors including economic growth and development, but also the globalisation of unhealthy lifestyles, unplanned urbanisation, population growth and population aging ([6]: 5). The global politics of addressing the NCD crisis, however, are puzzling and one could argue that we are only just beginning to probe these in some depth, in search of a more nuanced understanding of the mismatch between the enormity of the crisis, the broad range of commitments made by states and the insufficient actions relating to these commitments.

A number of leading analysts have shown a strong preference for the development of new international legal agreements as a means of reducing the incidence and prevalence of NCDs globally [23, 29, 47–50]. This view is shared by the United Nations (UN) and some of its agencies (e.g. the United Nations Development Programme (UNDP) and WHO), which have consistently advocated the creation and implementation of international legal agreements and other legislative measures in relation to NCDs (*see* for example UN [82, 83, 87–89]).

Some concerns have been raised with the adoption of a legal approach to NCDs, including the need to consider some regulation of the structural determinants of NCDs by including strategies for influencing broader socio-economic factors, poverty, underdevelopment and inequalities [9, 19, 69, 96]; the evident difficulties of implementing FCTC [56, 78]; the concern that a legal approach might be hijacked or diluted by corporate interests resisting regulation [12, 13, 77]. The WHO report *Time to Deliver* notes further concerns around effective prevention and control of NCDs, where challenges to implementation of existing policies and action plans include lack of political will, lack of technical capacity, insufficient policies and plans for NCDs, difficulties in priority setting, insufficient financing, lack of accountability ([92]: 12). Evidence from empirical studies in various fields of international relations and law directly and indirectly supports the position of those who have expressed scepticism that a new international legal agreement is indeed the most effective mechanism to curb the global pandemic. Drawing on existing conceptual and empirical knowledge of norm development and critical legal studies, this article argues that a new international legal agreement is not only improbable in the short term, but may also not be strictly desirable.

Analysts promoting ‘practice-oriented’ and ‘agent-focused’ approaches in international relations analyse normative change at the intersection of politics and law and propose conceptual models, also referred to as ‘norm life-cycle’ models of normative development and change, and apply these to empirical studies of the political dynamics of international norm development across a broad spectrum of issue areas in international politics. Insight will be drawn here particularly from the work of ‘agentic constructivists’ [71], with their focus on practice and agency in international politics and the emergence of new international norms. Insight from these studies and models is directly relevant to discussions promoting the development of international law as a mechanism for curbing the lifestyle disease pandemic, as they not only highlight patterns of political activity, but also reveal some less obvious and often overlooked aspects and dynamics of international normative development and change. Three insights in particular are of relevance to this discussion - the nature of the norm development process (long-term, contingent and unpredictable); the significance of the way in which the problem is constructed (framed); the opaque, but significant influence of corporate interests.

For the purpose of this discussion, the term ‘norm’ will be defined as ‘prescriptions for appropriate and acceptable behaviour from which the standards of behaviour are further negotiated and institutionalised’ ([75]: 13). Norms can remain unwritten prescriptions for appropriate behaviour but can also be developed further and embodied into legal principles. Depending on the level of codification, legal principles can range from ‘soft’ to ‘hard’ law, where hard law is most often expressed in the form of international legal agreements/treaties, while soft law is seen as ‘those nonbinding rules or instruments that interpret or inform our understanding of binding legal rules or represent promises that in turn create expectations about future conduct’ ([26]: 174).

Critical legal approaches also examine practice and agency in national and international politics as they relate to law more generally and to individual legal principles. These approaches draw attention to some of the drawbacks of a legal approach. Admittedly, these approaches are diverse and scholars differentiate their focus and assumptions within the broader approach, but there are a number of recurring themes, which make their insight relevant to this discussion of creating international legal instruments for the prevention and treatment of NCDs. These critiques of law dovetail closely with observations of the practice of developing new international norms. In particular, critical legal approaches call for awareness of and consideration for the deeply political nature of law, which is often reflective of prevailing societal power hierarchies and the political dominance of particular social, political and economic interests. As a result, international law and norms, it is argued, can exacerbate problems of underdevelopment and marginalisation and increase disparities in health between rich and poor ([18]: 14–15). Critical legal approaches commonly promote the view that legal doctrine (or legal instruments) is indeterminate in character, and therefore neither inherently good, just, nor fair.

The argument of this article will be set out in three sections below - the first section will outline the main premises of the agentic constructivist literature, highlighting the importance of understanding the *process* of normative emergence and change. It will further draw on empirical examples from different fields of global politics to illustrate some of the often-overlooked aspects of normative development. The second section will engage with the critique of legal *principles* as codified embodiments of norms*.* Critical approaches to law - both national and international - emphasise the pathologies of the national legal process, as well as the implications for legal principles, exposing the myths that legal principles are neutral, objective, good. The third section will discuss the characteristics of *practice* in the NCD field and implications emerging from these in the context of the discussion of process and principles.

In conclusion, it is proposed here that further in-depth critical analysis of and reflection on the strengths and weaknesses of a legal approach to addressing the NCD pandemic is needed. Such analysis ought to factor in the global politics of NCDs, including the interplay between public and private interests, the power dynamics between public and private actors, take a broad view of the social determinants of NCDs and seek effective interventions at individual and societal level. Such analysis is needed in order to consider what might constitute effective mechanisms and approaches to mediating NCD morbidity and mortality for everyone, but particularly for those most at risk in a dynamic global system, defined by profound inequalities.

## Main text

### What do we know about the norm development *process*? - insight from agentic constructivism

‘Agentic constructivism’ - a branch of social constructivism in International Relations (IR) [71] has dedicated a significant amount of research to international norms. Agentic constructivist work is both conceptual and empirical. Conceptually, the three most significant contributions of agentic constructivists are - firstly, the *foregrounding of agency* in contrast with traditional approaches to international relations, which tend to preference structure and the agency of the state in international relations. Agentic constructivists interrogate an often-overlooked area of international policy-making - the interrelatedness of agency, identities, interests and ideas in world politics and illustrate conceptual discussions with empirical research on the roles that agents play as drivers of international politics [3, 4, 16, 27, 37, 41, 60, 61, 70]. Secondly, agentic constructivists propose *models of norm development* that conceptualise recurring patterns of practice relating to the emergence of new norms – from the inception of an idea all the way to the codification of norms into legal principles and agreements [17, 64, 75]. This highlights the significance of thinking about norms and legal principles as an integral part of international politics, and not simply as an element of the broader social context. And thirdly, by combining the foregrounding of agency and studies of practice, agentic constructivists draw attention to *change* in international politics, where change is illustrated by the construction of new norms, evolving institutional mandates and practices (*see* for example [4, 72]).

These discussions take place in the context of broader disciplinary debates around agency and structure, change and practice. The arguments made by agentic constructivists are neither entirely novel, nor unique – parts of these arguments appear in critical and poststructuralist analysis of international politics. The agentic constructivists’ contribution is the focus on the politics of international norms and their relationship with state action by looking at international norms’ emergence, relevance and influence, which make their work particularly relevant to this discussion. Agentic constructivist analysis goes against the grain of mainstream theories of international relations ontologically. Where mainstream theorists consider the principles governing international politics to be largely ahistoric, static and immutable over time, subject to the goals of the pursuit of power and security, constructivists see norm emergence, change and context – historic (temporal), geographic (special), political, cultural, economic, etc.

Empirical studies of norm development offer a framework for thinking about the emergence of international norms, draw attention to the role of agents driving normative change – through campaigning, resistance, action, advocacy, negotiation, etc., to the varying forms of power employed by agents in pursuit of such change, to the role of normative networks bringing together a diverse array of actors in the norm development process, to examples of stumbling blocks in negotiations and so on. In his discussion of framing of normative ideas, however, Payne draws extensive attention to the contingency and unpredictability of such processes ([59]: 43–47). The role of contingency is also evident in other constructivist studies of the evolution of norms (*see* [60, 75]). Expectations, therefore, that generalisable conclusions, based on the experiences of emergence of past norms can be drawn to inform or direct advocacy on NCDs in the future or that analysts can identify critical factors determining the success or failure of law-making efforts, as hinted by Toebes et al. [80], are unrealistic.

Constructivist research on norms was never intended as a tool to predict norm development, to provide recommendations about how to influence international policy-making, or indeed to prescribe particular content or format for new norms. The factors influencing each norm development process, the networks of actors that form, the way these actors make the case for their vision of a new norm are deeply unpredictable, contingent, idiosyncratic. Observations from individual empirical studies are not directly generalisable, due to the complexity of political and social relations, temporal, spatial and normative contexts, the vast array of factors that impact norm emergence and so on. Agentic constructivists’ tentative generalisation about norm development, therefore, is useful in thinking about the global politics of noncommunicable diseases in two ways – conceptually, as it provides a framework within which the politics of NCDs in pursuit of a global norm(s) can be traced and interrogated, and empirically, by examining patterns of norm-related advocacy and practice across a diverse range of examples studied by constructivist scholars.

One aspect of agentic constructivists’ conceptual contributions stands out as directly relevant for the analysis of the politics to address the NCD challenge - namely, the norm development model as presented by Stoeva [75] in *New Norms and Knowledge in World Politics*, building on the work of Finnemore and Sikkink [17] and Risse et al. [64] among others. The model comprises seven ‘stages’ – initial idea, issue formulation/framing, network configuration, dialogue between supportive and conservative states, political closure (if persuasion succeeds), legalisation/codification, operationalisation ([75]: 26). The model is not intended as linear and the stages are not chronological. In practice, the stages can and do overlap, the process loops back to previous stages and can also end at any stage. The stages of the model indicate critical moments of change – the emergence of a normative idea, the framing of a problem, the pursuit of normative and technical, then political closure, etc. and processes that are indicative of norm emergence – changes in rhetoric, changes in practice, negotiations, codification, etc. The norm development process consists of multiple and concurrent processes of knowledge construction, advocacy, coalition building, argumentation, negotiation, coercion, bargaining, consensus building, political lobbying, and so on [17, 59, 63, 75].

Empirical constructivist analyses highlight that the outcome of a normative campaign is never guaranteed. Outcomes are shaped in the process of negotiations, as well as through lobbying, bargaining, argumentation, coercion. Factors that are often overlooked, but can be significant in shaping the outcome of norm development campaigns include personalities of political and organisational leaders (as the campaign for a Convention Against Torture illustrates - [75]:52–53, 55), the way in which a problem is framed (as illustrated by the normative campaign on landmines - [60]; or intellectual property rights - [75]:86–93), broader public attitudes, the nature of other issues competing for the attention of policy-makers, linkage bargaining, and other factors that are impossible to predict.

Three themes emerge from empirical constructivist analysis that can be considered relevant to NCD politics. Firstly, studies show that the time frames for norm emergence are significant - often taking on average a decade for legal agreements to be negotiated – from political closure on the need for a new norm to signing of legal agreements; and more than two decades if we consider norm emergence from initial idea to political closure. Secondly, even though there is no formula for successful problem framing, the way a problem is framed and contextualised within the existing normative framework is of central importance - if a framing fails to generate support and political traction regardless of how significant a problem is, norm emergence is delayed or worse halted. Thirdly, corporate interests play a very significant, albeit often invisible role in international negotiations, as corporate actors often lobby governments in situ rather than at global forums, making their influence very difficult to discern and study.

Studies of the emergence of a broad range of norms - including the protocol banning the use of chlorofluorocarbons (CFCs) [27], norms condemning apartheid [40], the convention banning landmines [60], individual criminal responsibility [72], convention against torture, intellectual property rights, norms promoting climate change prevention [75] - provide empirical evidence that the process of conceiving, framing, constructing and negotiating new norms is long, complex, contingent and indeterminate. In most of the above examples, it took over two decades for networks of norm entrepreneurs to convince states actively opposing or not supporting a new norm to concede to it and lend their consensus, and longer still for that norm to be codified into an international convention. Further time was needed for an international treaty or convention to be ratified by state signatories and implemented at the national level, to negotiate optional protocols or create institutions to facilitate and oversee implementation and compliance. The norm emergence process covers the full spectrum of activity from the inception of a normative idea through to legalising, institutionalising and operationalising an international norm by embodying it in an international agreement (if the norm gets to that stage). Contestation and resistance accompany norm emergence at every stage. Even after norms have been agreed, implementation and compliance are not guaranteed. So much so, that the latter are emerging as separate fields of research, to examine the specific political and economic dynamics associated with these processes (*see* for example [10, 11, 65, 73]). As noted by Yach [94] it took over 50 years between medical professional reporting on the health consequences of tobacco use and the adoption of the FCTC. Civil society actors have been lobbying for international treaties on alcohol and healthy diets for over a decade and a half. This significant time lag between campaigning for a new norm and codification (whether as soft or hard law) needs to be taken into account by advocates of a legal approach to the NCD crisis, in which state action and attention are urgently needed. A decade or more is a long time when seeking to promote action on an issue responsible for 70% of all deaths worldwide.

An early and crucial stage of the norm development process, as set out by Stoeva [75] is ‘issue formulation’. This is a multifaceted process that often takes place simultaneously with the consolidation of advocacy networks for normative development/change. The process of formulating an issue involves defining and framing a problem in normative terms - i.e. reaching agreement on the normative reasons why something is a problem, defining causes and effects in technical/causal terms, and framing the problem in a way that fits in with existing normative frameworks and contemporary political agendas to ensures that the problem is addressed most effectively and without delay. The significance of this process cannot be overstated. Its complexity and unpredictability, however, is significant - multiple actors with diverse identities, potentially driven by divergent interests, with access to varying resources, seek to influence policy-makers in a dynamic global and domestic context to achieve the normative change that they are pursuing. Payne [59] discusses framing in the process of normative change with reference to the campaign for creation of ‘core labour standards’. He concludes that: ‘[p]olitical contexts are often highly contested and it can be essentially impossible either for norm entrepreneurs to know in advance which frames might work or for scholars in retrospect to ascertain the resonance of any particular frame or counterframe’ ([59]: 52). Payne also warns that ‘rhetoric can be manipulated to seem reasonable for audiences… [and] frames must be understood in terms of prevailing power structures’ as well as that coercion rather than persuasion is often involved in the norm development process ([59]: 54). To him, this undermines the legitimacy of international norms, which is a conclusion that resonates with some of the work of critical legal scholars as discussed in the next section.

Price [60] also highlights the importance of successful framing in the case of banning the production and use of landmines. Before the successful civil society-led campaign that led to the creation of the international convention, there was an unsuccessful campaign to outlaw the use of landmines, framed as part of conventional weapons disarmament treaties ([60]: 618–9). Intellectual property rights were initially loosely regulated by the World Intellectual Property Organisation (WIPO) until the abuse of intellectual property through piracy, counterfeiting and free-riding on United States (US) inventions was framed by a group of large US corporations as one of the leading causes of US growing trade deficit, overall economic decline and significant loss of corporate profits ([75]: 86–88).

Framing is a of central importance for any campaign for a new norm relating to NCDs as well. As noted by Bogart [7] in relation to questions about obesity, framing is not only complex in its own right with extensive possibilities to get things ‘wrong’, it is related to having a broader in-depth understandings of possible solutions and monitoring the evidence of what works. Gneiting and Schmitz [22] offer in-depth analysis of the differences between the framing of the problem of tobacco and alcohol use, highlighting various factor that have impacted on framing and the far-reaching effects these have had on the normative campaigns. The implications of an uncontrolled NCD pandemic have so far been framed in terms of them being detrimental to economic growth and development [5, 55, 58, 83]. Proposal for alternative framing of NCDs include taking a human rights approach to NCDs (e.g. [14, 25]) and framing NCDs as a health security challenge [31, 67] in search of impact and political attention.

The significant and broadly understudied role of corporate actors in global politics and global health governance is the third insight from agentic constructivist research to be discussed here. Corporate actors are a particular type of social entity. They are usually privately-owned, set up to produce a commodity or a service with the overarching aim of making a profit. Global corporate actors wield a significant amount of power in economic, social and political relations. In the process of production and profit-making, corporations also make local investments, employ people, pay taxes; sometimes they damage the natural environment, but other times invest in improving local infrastructure, and so on. Their activities have diverse effects on governments and communities. And while mainstream IR theories often put governments and corporate actors in separate categories and define them in opposition to one another, in practice, the interests of society, individuals, governments and corporations are much more intricately intertwined - aligning and diverging on different issues. Global corporate actors occupy a unique space in political relations. They wield significant material power through finance, resources and the ability to invest. They often have unrestricted access to policy-makers and the policy-making process and are often actively consulted in the latter through a variety of formal and informal channels. Their influence is difficult to study because it takes place away from the public scrutiny.

Corporate actors play an active role in international negotiations. Contrary to conventional wisdom, they are not always opposed to international norms - corporations may actively lobby for a particular norm – e.g. the insurance industry in the case of climate change or the pharmaceutical, automobile, chemical and tech industries in the case of intellectual property rights protection [75]. Corporations can also actively participate in devising solutions to a given problem - e.g. DuPond in the case of regulating the use of CFCs [27] or the diamond industry in the Kimberley process [24, 93]. More traditionally they are known for seeking ways to block or undermine an emerging norm as does the non-renewable energy industry in the context of climate change negotiations [75] or the tobacco industry [13], the food and alcohol industry [12]. What we know with some degree of certainty is that corporate actors are always driven by a logic of consequences, as their raison d’etre (in both economic and legal terms) is profit-making, and less so by a logic of appropriateness. As the corporate identity is primarily defined (even in legal terms) by the pursuit of profit, corporations can ‘get away’ with prioritising utilitarian concerns for profit-maximisation, at the expense of ethics, justice or sustainable development, in a way that other actors – e.g. civil society organisations, states, intergovernmental organisations - cannot. Consumer pressure and attempts at self-regulation has in the past made it appear that corporations act in accordance with ethical principles, but there are few if any structures, norms or mechanisms transnationally that can make powerful multinationals comply with international legal norms. States are too often not capable of controlling corporate activity in a way that they can control the activity of other private agents.

Empirical studies demonstrate that corporate power is pervasive and extensive in its attempts to deter the development of health policy. Through domestic lobbying, coercion, litigation and other mechanisms for political and economic pressure, corporate actors more often than not manage to get what they want (for studies of the influence of the tobacco industry on policy development*see* e.g. [13, 85, 86]; for studies of the resistance to health policy of the alcohol industry *see* e.g. [51, 53, 68]). This is almost a consistent observation of analysts across a broad spectrum of issue areas and a consideration to be borne in mind by advocates of new NCD norms. The corporate power of the tobacco, alcohol and food industry is potentially commensurate with, albeit different in character from, that of the pharmaceutical industry, the automobile industry and research has shown the profound impact that these industries had in shaping the global intellectual property rights regime in their favour. Policy makers, civil society and intergovernmental organisations need to seek ways to counter-balance corporate power in the global politics of NCDs.

In view of these observations and agentic constructivist research, it can be concluded that legal norms (whether national or international) should not be perceived as panacea to societal problems. Rather, they are mechanisms aimed at responding to issues that have been constructed as problems, to which a sufficient number of states has agreed to respond in a particular way, which is deemed normatively appropriate (given social rules) and technically effective (given the laws of science) ([15]:71). The agentic constructivist conceptualisation of norm emergence as process-oriented aims to ‘denaturalise’ state agreement on normative principles and demonstrate that such agreement is a product of the complex interplay of multiple actors, interests, identities, institutions, practices, existing norms, relationships of unequal power, etc. Such a conceptualisation of international norms is consistent with views expressed by the ‘process’ school of thought in international law and by critical legal scholarship ([8]: 79–81). These views stand in contrast to the dominant conceptualisation of international law as outcome-oriented, where law is often perceived as an instrument to achieve particular goals or values (e.g. human dignity, democracy, gender equality and so on) and its shortcomings are evaluated in relation to the achievement (or lack thereof) of these values ([8]: 82–84). A process-oriented approach provides a framework for analysts to examine the broad spectrum of influences shaping the emergence of international norms.

### Are legal *principles* the most appropriate mechanism? - views from critical legal scholarship

There is substantial advocacy for the development of international legal principles (whether ‘soft’ or ‘hard) almost to the point of assuming that international law is the most appropriate instrument to address the global NCD pandemic [23, 29, 48, 49, 57, 79, 80]. As noted earlier, this approach is broadly supported by the UN and its agencies. International legal principles or agreements are often perceived by proponents as the most effective vehicles to implement required action or pursue desirable outcomes in the external relations of states. They are viewed as symbolic of the commitment of states to work together in the pursuit of common goals such as peaceful coexistence, certainty, predictability, order (and even justice), as a commitment to inter-state cooperation and the avoidance of war ([30]: 13–16 [46, 74];).

Critics put such assumptions under a microscope, drawing attention to the inherent indeterminacy of law and the ability of lawyers to selectively use existing legal principles to make whatever case they are seeking to support ([42]: 354). Attempts to de-naturalise legal principle and depose law from its idealised pedestal are consistent to a degree with agentic constructivist findings that emergent legal principles are constructed within particular contexts, normative frameworks, institutional architecture, and most definitely in the context of prevailing power hierarchies. Having said this, constructivists, also tend to (uncritically) embrace the emergence of new norms as a positive development in international politics.

It is proposed here that some caution needs to be exercised in such assumptions. While an international treaty or a convention setting out a clear agenda for global concerted action may sound as the missing piece in the politics of NCDs, in practice, international law is rarely the main factor driving political action, even less so in driving successful and long term change in values, patterns of action, behaviour or consumption broadly speaking. Some constructivists go so far as to argue that law is instead itself the product of such changes (*see* e.g. [72]). In other words, the hope that advocating for new law would lead to changes in values or behaviour is fallacious. What is needed instead, and I will return to this point in the next section, is an underlying commitment by states and other actors to engage with the prevention and care of NCDs – an international legal agreement, if one ever materialises, is only one tool in a much broader political toolbox of instruments to pursue effective action. Below is a brief discussion of the critical legal tradition highlighting some of the deficiencies of law as a policy instrument.

The critical legal tradition is divergent with some branches or approaches much more extensively developed than others. Thus, for example, Critical Legal Studies (CLS) emerged in the United States in the 1970s as a substantive, in-depth critique of American jurisprudence including both legal principles (doctrine) and legal practice. In contrast to the critique of a national legal system, critical international legal theory (CILT) is a broad collection of critical legal thought focused on public international law. CILT is underdeveloped and less structured in comparison with CLS and the former is by no means an extension of the latter [36]. Far from offering a detailed account of critical legal studies or critical international law theory (including feminist thought, post-colonial and post-modern critique), this section will draw out some of the core criticisms that proponents have levied against the dominant liberal legal order, which are relevant considerations in advocating appropriate mechanisms to address the unfolding NCD crisis.

CLS scholars have produced a substantial body of legal criticism of law in modern Western society, but primarily in the US (for a more detailed discussion *see* [34, 66, 81]: 2). Some of the core elements of this critique can be summarised as follows: the implicit oppression of this order, which obscures the origins of legal doctrine and legal decisions, in a society defined by power differentials, skewed in favour of the white, upper-middle class males who are in a position to manipulate legal rules to reflect their ideology and to normalise/naturalise doctrine and legal decisions ([66]: 13–14 [2];: 15). CLS critiques the pretend objectivity of the liberal legal order and scholarship for its assumptions and pretence that the world is rational, necessary and just rather than arbitrary and contingent ([35]: 210–1). CLS further notes the even more devious side of the liberal legal order - the legitimation of unjust social relations by making them appear either necessary or desirable ([66]: 14 *citing* Rubek, Unger, Kennedy). Liberal society, therefore, in the eyes of CLS is ‘riddled by domination and hierarchy [where] class and managerial elites set the terms upon which others are to lead their lives’ ([35]: 209). This action of denaturalising the social and legal order, pointing out that its dynamics are a result of differential powers at play, that decisions (even legal ones) are choices, rather than inevitable doctrine, that legal principles and process do not operate outside of all other social and political relations, creates space not only for critique of the existing order, but also for seeing this order is one possibility among many.

To the extent that we can talk about recurring themes, both CLS and CILT highlight the political nature of law and legal decisions, underpinned by the inherent indeterminacy of law ([2, 38, 43]; for a more detailed discussion of CILT *see* [39]). This emphasis on the political nature of law and legal decisions highlights that there is nothing in the legal system that guarantees either the emergence or the practice of law that is fair and just to all. Koskenniemi argues that international legal language is both strict and indeterminate, in the sense that it is both ‘rigorously formal’ while also allowing the defense of conflicting positions ([42]: 354–5). Other critics go so far as to state that ‘... the very idea of the rule of law serves as an instrument of oppression and domination’ (Altman 1993: 15). Critical legal scholars warn us that every aspect of the law - from its process of creation, to its rules and their interpretation and application is a ‘political act’, and what defines the political in political science is its focus on studying relations of power. In other words, taking a critical view to legal rules allows us to ‘see’ the politics in process, principles and practice. Law, in other words, cannot and should not be seen as independent from politics and political hierarchies, but rather as an instrument used by said hierarchies, to ‘make particular intellectual or social hierarchies appear as natural aspects of our lives’ ([44]: 16). Such practice embodies the twin actions of reification and legitimation, which solidify and naturalise the foundations of a particular and largely unjust legal order, which naturalises contemporary hierarchies and perpetuates the silencing and marginalisation of poor and less fortunate individuals and communities.

Critical views of law prompt us to consider international norms from a different angle and encourage us to foster an awareness of the way such norms affect different states, communities and individuals. This is not to say that law cannot or should not be used as part of the approach to curbing the global NCD pandemic, but that its utility and appropriateness and embeddedness in a system of profound power inequalities ought to be critically evaluated. Law can be used for example to affect structural determinants of health and promote joined-up cross-sectoral policy, or as an instrument creating a framework for action, supporting states in taking on corporate power. Norm advocates, therefore ought to evaluate whether and how international law can deliver regulation, which does not itself become an instrument of oppression of the weaker, voiceless and marginalised states and communities within states. If law cannot achieve this, then alternative options ought to be considered – such as for example policy responses based on broader consultation and consensus, bottom-up community-based initiatives, alternative mechanisms that can put pressure on corporate actors. Learning from other fields of global policy-making, further research and some ingenuity is needed to consider viable alternatives.

### What does this mean for the *practice* of global prevention and control of NCDs?

Applying insight from studies of norm development and from critical legal analysis to the politics of curbing the global noncommunicable disease pandemic provides a useful lens on normative processes and principles. On the one hand, it enables a more nuanced, practice-oriented understanding of these politics and on the other, it prompts critical reflection on the principles that are being advocated. Much like other global problems, NCD morbidity and mortality disproportionately affects low- and middle-income countries, as well as the poorest and often most disadvantaged strata of societies in high-income countries [33]. Prevention and treatment of noncommunicable diseases has often been described as uneven, inconsistent, under-resourced, compounded by inadequate and/or inequitable access to care and essential medicines, but also most problematic in the same regions and societal strata. Despite the adoption of an international treaty (FCTC), a number of UN High-Level Meeting political declarations, WHO action plans and a wide range of national legislation to address the modifiable behavioural risk factors associated with NCDs, there is broad agreement that not enough is being done to address the crisis [20, 31, 32, 91, 92].

The discrepancies between the urgency highlighted by analysts and advocates, the high levels of NCD-related morbidity and mortality globally, but specifically amongst the poorest states and communities, the inertia amongst policy makers, but also the ways in which existing norms and principles are affected by harmful power hierarchies, and whether the former do or do not address poverty, inequalities and underdevelopment both as causes and consequences of the NCD crisis, need further reflection in the process of devising and advocating potential solutions and policy options. This section will briefly discuss the characteristics of practice and the politics of NCD, and the implications emerging from these in the context of the preceding discussion of insight from agentic constructivism and critical legal studies.

#### Norm emergence

Agentic constructivists note that campaigns for new norms tend to follow disasters or crises on a significant scale, where norm entrepreneurs are willing to drive forward a campaign for action ([84]:35 [75];: 25). Empirical studies of norm development suggest that more often than not norm entrepreneurs are individuals, advocacy groups and/or civil society organisations - such was the case for the Treaty banning the use of Landmines and the Convention Against Torture, even global climate change (*see* [60, 75]: 44–50, 126–131). In some cases corporate actors can also drive the idea of a new norm forward, as was the case for the regulation of intellectual property rights ([75]: 86–88). In all situations, however, norm entrepreneurs work closely with governments willing to take particular ideas forward. Studies of NCD-related normative campaigns tend to acknowledge but not necessarily discuss the role of civil society organisations at great length. Much more attention is focused on corporate interests, as they are strongly opposed to regulation, and of activities undertaken by corporations to undermine regulation efforts [12, 28, 45, 54]. As illustrated in other cases, such corporate activity has the ability to slow down international negotiations, undermine the strength of emerging norms, or even prevent new norms from being established. Studies of the food and alcohol industries demonstrate that there is little interest among these actors in supporting stringent product regulation and that these industries have lobbied heavily for voluntary self-regulation, which has been shown to rarely deliver meaningful results. All of the points raised so far, therefore, put the chances of the emergence of a strong coalition to play the role of a norm entrepreneur quite low.

There are two further fundamental problems with the NCD normative campaign. Firstly, there appears to be no agreement on the best way to frame the problem, which requires solution. Current framing does not seem to be able to purchase the traction needed to get NCDs to the top of the global political agenda – a spot that many an issue are competing for – climate change, conflict, communicable diseases, antimicrobial resistance, humanitarian and refugee crisis, multiple security hotspots, etc. Secondly, and related to that but separate from the question of framing is that of the way in which the cause of the problem is defined. There are two broad approaches, on which policy advocacy has been based - the individual and the structural approach [95]. The individual responsibility thesis, often promoted by industry, but supported by states and analysts, largely focuses on individuals needing to take responsibility for his/her behaviour and choices. The structural thesis revolves around broader determinants of health - ‘[w]hat we describe as a *structural* approach to NCDs focuses on enduring social arrangements that determine the pattern and distribution of NCDs and their risk factors in society’ ([95]: 2). Which approach is preferenced by policy makers will largely determine the point and nature of intervention, the action required to address the perceived cause, the onus of responsibility. Clear definitions of the nature of the problem - in terms of identifiable causes and effects, evidenced empirically (as was the case for the convention against torture [75] and the ban on CFCs [27], along with effective framing of the problem as illustrated by the case study of the campaign against landmines [60], and intellectual property rights [75] have in the past made for successful normative development campaigns. One very prominent example - the case of the campaign to avoid catastrophic climate change [75] demonstrates how the lack of agreement on the nature, causes and effect of a problem can undermine an otherwise strong normative campaign, resulting in a weak international norm.

Normative closures are often difficult to achieve, as they are premised on a clear definition of a contained problem, which conflicts with established norms and broader understandings of appropriateness. Technical closures tend to revolve around scientific knowledge and solutions premised on causal relationships. Analysis of NCD politics has noted issues with the leadership of civil society organisations working on NCDs [19] and with corporate actors being represented at UN civil society meetings [12, 77], which is significant, because civil society organisations are usually the actors at the heart of normative coalitions. This is counteracted by strong industry interests driving a particular understanding of the causes of non-communicable diseases - namely, defined as individual choices in a free market economy. In the case of NCDs, however, while the industries that will be affected by future regulation broadly share an interest in keeping such regulation weak and voluntary, they are also likely to be affected differently by such regulation – e.g. regulation of the food industry is likely to require it to change its products rather than restrict or reduce the use of its products as is the case for the alcohol and tobacco industry.

Some critical social science work is beginning to emerge, analysing the politics of non-communicable diseases in the global South, which can be of great value to advocates of norms that are inclusive and equitable, but also applicable in a variety of different contexts (*see* for example [21, 62]). This work needs to be utilised effectively to counter-balance the strong tendencies towards framing solutions to the problem of NCDs purely as the need for behavioural interventions in response to the four identified risk factors of tobacco, alcohol, diet and physical inactivity. In the context of framing the problem and as evidenced by normative campaigns in other fields, further issues that advocates for an NCD-specific international norm need to be aware of include the relationship of such a norm with already existing norms, particularly those in international trade, so as not to leave states open to litigation from corporate actors ([90]: 11 and 21), but also norms around universal health coverage (UHC) and the right to health. Identifying the way a new norm fits within and enhances the existing normative framework can contribute to a more persuasive normative campaign and to states more readily agreeing to pursue international agreements. Thus, building on the consensus on UHC and the advocacy and practice of the right to health, a treaty on NCDs may be easier to negotiate when linked to these norms.

#### International law - one amongst many approaches

International law is premised on an outdated state-centric paradigm while operating in a world where states no longer enjoy absolute sovereignty or power. A key deficiency of international law is that it applies directly only to states, and only indirectly to non-state actors - including multinational corporations (MNCs), which makes it very difficult for individual states to govern these entities. As previously noted, MNCs wield an enormous amount of power over governments and communities - not only through the resources that they have at their disposal, but also through their power to disinvest or relocate - threatening jobs, local investment, trade and tax revenue. Creating a new type of global regulatory regime capable of countering corporate power at least in part, is a task of a very high order.

A number of proposals have been put forward by analysts about possible action, offering a range of possibilities to governments and advocates of change. There is a strong emphasis, for example, on a comprehensive approach to the problem of NCDs that addresses individual choices and behaviours along with structural inequalities, social and economic determinants of health [5, 9, 52, 76, 95]. While this would be one way to ensure the best of both worlds, it may not be possible to combine these two approaches within the same regulatory instrumentarium. Considerations need to further be given to other mechanisms to promote action, which can produce more immediate results in the short to medium term – including bilateral, multilateral and regional cooperative efforts by states, soft law commitments by states, but also grassroots activism, trans-local and national level action and agenda setting. Analysts ought to evaluate the viability of such proposals - both conceptually and empirically. In considering policy recommendations and approaches, scholars and analysts become activists in the promotion of specific policy solutions to the NCD crisis, and it is important to consider both the ethics, as defined by a logic of appropriateness and the technicalities as dictated by a logic of consequences, of proposed solutions, as these are the languages of normative development and the basis for pursuing political closure. As academics, we are responsible for advocating inclusive and ethical norms, but also for raising awareness of the implications of inequalities and differences between and within states and societies.

To be effective and increase the chance of having an impact on policy-makers, as demonstrated by empirical research, communities of scientists (aka epistemic communities) need to reach out to broader networks of activists and norm entrepreneurs. It is of course necessary to remember that political processes are inherently indeterminate, contingent, organic, open-ended, influenced by practitioners, activists, policy-makers, industry, public opinion and a myriad of contextual factors. This means that once a normative process is underway no one actor is in a position to control the outcome, this includes corporate actors, even though they are in a strong position to exercise much influence on policy-makers, as previously discussed.

## Conclusions

Research on the politics of noncommunicable diseases is rich and expanding. Analysts are examining the field from a variety of different angles and there is no shortage of expert opinion not only on possible avenues for action, but also on specific programmes of action. There is significant advocacy for the development of an international legal agreement to stimulate and support action globally to address the noncommunicable disease crisis. This article sought to bring insight from the fields of international relations and international law to bear on such advocacy and highlight potential pitfalls of a legal approach to the politics of NCDs. Drawing on conceptual and empirical research by agentic constructivists, a number of observations were made - around the typical time-frames of norm development; the central importance of appropriately and effectively framing the problem(s), which require regulation in a way that is current, relevant and pressing and manages to sustain policy-makers’ attention; and the concern about the role of corporate influence in global negotiations, particularly when their interests are directly at stake. These observations based on empirical studies of the development of other norms are corroborated by analysis in the NCD literature, meaning that they are of significance to this particular norm development process as well. Initial analysis, premised on the model of norm development, demonstrates that the process of developing a new international norm on NCDs is still very much at a nascent stage. Using this model has highlighted areas that are still open for advocacy and others that require strong input to keep potential future normative negotiations on course to maintain a strong equity focus in line with other global health norms.

The article then discussed contributions of critical legal scholars, whose observations about the nature and character of law in general and international law in particular align with the empirical research of agentic constructivists and raise important questions about the desirability of adopting a legal approach to the politics of NCDs. These scholars are critical of the influence of unequal power relations on both legal doctrine and practice, and are concerned about the way in which law is often portrayed as both the best possible mechanism to guide behaviour and a product of the natural evolution of political discourse, thus obscuring the politics of law, laws’ subjectivity, historicity and dynamism. These contributions were introduced to provide a counter-argument to mainstream advocacy for legal mechanisms to address the problem of non-communicable diseases for current and future generations. Critical legal scholars problematise and de-naturalise law, suggesting that other orders are possible, and that legal mechanisms can be designed differently or other mechanisms may be more effective altogether if the flaws of legal mechanisms are too profound.

The combined criticisms and caution expressed by critical legal scholars and observers of norm development raise important questions - e.g. whether international law offers the most desirable approach or the most effective mechanism to address the global NCD crisis. Short of radical new ways of global concerted action, possible ways to counteract some of the problematic characteristics of international law as a policy instrument - such as its bias towards powerful interests or its indeterminacy - may be to demand more inclusive, representative and transparent negotiations; to support activists and civil society organisations from the Global South to attend and participate in negotiations; for advocates of a new norm to use carefully crafted language; to promote cross-sectoral rather than siloed negotiations that evaluate the combined influence of existing international norms; to consider the resources available to commit to operationalising potential new international agreements; and commitments to secure assistance with implementation.

The estimated opportunity cost of inaction is £30 trillion over the next 20 years [6] and that does not account for the human cost in terms of poverty, individual suffering, underdevelopment, insecurity and so on. In view of the potential time frame of developing a new international agreement and the time lag of legal principles being put into national legislation or other forms of action, it may be more effective to discuss concerted actions in terms of ‘soft’ rather than ‘hard’ law, or series of actions for the short- and medium-term, while such a norm is negotiated. There already exists an overarching normative framework to support such programmes of action - the right to health, commitments made by states to deliver universal health coverage, UN political declarations on NCDs, WHO Action plans, and crucially the Sustainable Development Goals.

## Data Availability

Not applicable.

## Abbreviations

CFCs: Chlorofluorocarbons
CILT: Critical International Legal Theory
CLS: Critical Legal Studies
FCTC: Framework Convention on Tobacco Control
IR: International Relations
MNCs: Multinational Corporations
NCDs: Noncommunicable diseases
UN: United Nations
UNDP: United Nations Development Programme
US: United States
WHO: World Health Organisation
WIPO: World Intellectual Property Organisation
